# Supplementary material for: Development of a perioperative thermal insulation system: Testing comfort properties for different textile sets
Source: PLoS One. 2023 Sep 12;18(9):e0291424. doi: 10.1371/journal.pone.0291424 (PMC10497167; doi:10.1371/journal.pone.0291424)
Supplement: Attachment — Submitted filename: Rebuttal_ letter16 outubro.doc [file pone.0291424.s001.doc]

**Rebuttal letter**

Dear Professor Rajagopalan Parameshwaran,

Academic Editor of PLOS ONE

We are resubmitting the manuscript “Development of a perioperative thermal insulation system: testing comfort properties for different textile sets” for publication in PLOS ONE. We would like to thank you and the Reviewers for all the comments and suggestions on the manuscript and for the opportunity to review and resubmit this work. The comments and suggestions have enhanced the quality of this manuscript. This rebuttal letter shows the changes made and the explanation for each item. The added text is highlighted in yellow.

| **Editor comments** |  |
| --- | --- |
| lack of clarity in demonstrating the need of the study, parametric specifications and their significance for the system under consideration, the method of evaluation with adequate justification and the data available in the manuscript are not fully convincing. | All the manuscript was reviewed, and new background information was inserted in the Introduction, Method, Results and Discussion sections about reasons for conducting the study, the significance, and parameters of the system under development, the goals of the study and the evaluation method. Data were corrected and explained.  Since the manuscript was reviewed thoroughly and substantial changes were made, they are not copied here. |
| **Reviewer1** |  |
| The study investigated the thermal properties of inner layer fabrics to be used as garments for patients during surgery. Patients seem to suffer from low temperatures in operation theatres. However, no detailed evidence was provided in the manuscript about this issue. | Information was clarified in the article.  In the introduction:  1) The study investigated the thermal properties of the inner layer fabrics proposed for a three-layered blanket composed of three fabrics (one layer each).  2) Evidence was added to show the incidence, dimension, and significance of perioperative hypothermia. |
| it was unclear how the study should provide new insights for developing novel multi-layer fabrics for perioperative use | Information was added: “This study sought to combine existing fabrics to design a new blanket rather than developing new fabrics or materials. This innovative approach takes advantage of different fabrics and features: a three-layered blanket instead of three garments.”. |
| Some fabrics were selected as inner layers (without a rationale for the selection). These fabrics were combined with an intermediate and an outer layer (again, without further information if these are standard fabrics used in this setting). | Information was added justifying the selection of the fabrics and providing information about types of fabrics and systems used in the perioperative context. It was clarified that the mid and outer layers had been chosen previously and none of these fabrics are used in the perioperative context.  “The differences between layered clothing in sports activities and patients in the operating room, led to the following adaptations in structure and shape: (1) Use and combine existing fabrics, since the development of textile materials and fabrics is not an objective of this research; (2) developing a new blanket-type solution made with 3 different fabrics rather than layered garments for patients in the lying position; (3) a soft touch fabric used for the inner layer, a low density and highly porous fabric for the mid layer, and an impervious for air fabric for the outer layer; (4) breathability and perspiration of the outer layer would not be considered as important characteristics of this new solution, since the perspiration produced by the person during a surgery is incipient, contrary to the outer layers used in activities under adverse conditions that need to be breathable due to athletes perspiration; (5) a solution capable of mechanical washing and disinfection; (6) the lower cost of the fabrics for testing; (7) define the ease of penetration of hot air inside, its return to the patient and the reduction of the possibility of heat-escape to the environment were defined as the main characteristics of the solution. “  “Fabrics were selected according to the objectives of each layer. When considering the desired fabric air permeability [23,24] the fabrics for the outer and mid layer had been chosen and previously tested following the NP EN ISO 9237, using Textest FX 3300 Air Permeability Tester equipment with a pressure of 100 Pa and a test surface area of 20 cm^2^.  For the outer layer, the aim was to find a fabric that allowed air retention inside the blanket, preventing its escape to the environment and keeping the patient comfortable. An existing waterproof and slightly heavy fabric had been chosen, and its air permeability was tested. Results showed that the fabric was impervious to air with 0.3 l/m^2^/s.  For the mid layer, a fabric with a light, voluminous and low-density structure had been chosen and tested for air permeability, considering the need to create a considerable space that allows the accumulation of a cushion for circulating warm air without adding significant weight or volume. The produced results showed that the fabric for the mid layer was very permeable to air with 500 l/m^2^/s”  “For the inner layer, a friendly touch soft fabric was intended to choose. Three jersey fabrics were chosen with similar touch sensations.” |
| Benchmark tests were applied on the single layers, which differed between inner layer fabrics and intermediate/outer layer fabric (a thermal characterization of the inner layers without providing detailed information about the methodology applied and the calculation of the parameters; air permeability for intermediate/outer layer). Unfortunately, there was no explanation for why different tests were chosen for different layers. In addition, thermal manikin tests were conducted to test multi-layer systems. | Many arrangements were made, and excessive and non-relevant information has been removed.  Information has been clarified in the previous item. |
| Two different approaches were selected for the calculation of thermal resistance. Unfortunately, no information was provided on why both methods were considered and what kind of information can be extracted from one or the other approach. | Information was added in the method section: “The thermal insulation of the tested three-layered sets can be calculated in two ways: by adding the area-weighted local thermal insulation to the different body segments of the manikin - serial method - or by using the heat flow from the manikin´s body - parallel method”  “Serial and Parallel Models were used for this study. Although the Parallel Model is recommended for standard tests, in this study, both methods were used to understand the differences between them since the product being tested covered only half of the segments of the manikin” |
| Data from benchmark tests and manikin were only compared descriptively (no statistical analysis was applied to the data). | Proper statistical data were added. ANOVA analysis was performed, and a new table (Table 5) was added. |
| The discussion section is weak and does not consider findings from other studies but presents data from this study superficially (only one study cited in this section). | A major revision of the Discussion section was performed, and proper information was added. |
| Finally, the conclusion was drawn that using a three-layers set may be more suitable and provide more effective thermal protection than the other tested fabrics in patients during the perioperative period. This conclusion is not based on the experimental results from this study, as the effect of the inner layer fabric on the three-layered fabric system was investigated. | The conclusion section was rewritten to clarify some aspects and match the goals of the study.  “The thermal comfort properties of three-layered sets were tested to find the best thermal comfort performance, using a thermal manikin.  The set whose inner layer was composed of polypropylene, polyamide and elastane revealed the best thermal insulation levels, which indicates that the set is more suitable and provides more effective thermal protection compared with the other tested sets.  In view of these findings, the blanket will be tested in patients in the perioperative context, comparing its effectiveness for thermal comfort with the forced warm air system recommended by international guidelines.” |
| No information or data was provided about other fabrics/garments used for patients during surgery. | Information was added to this topic.  “Two types of body protection are commonly used in the perioperative context: passive measures or thermal insulation systems, which create a barrier to prevent heat loss from the patient's body to the environment - the most commonly used are cotton blankets; and active measures or warming systems, which warm the patient's body through heat from external sources.” |
| This manuscript lacks a clear structure and explanation of the study rationale and the goals. | A major revision of the article was performed. |
| The materials and methods section has to provide more detailed information about the calculation of the thermal properties of fabrics and the rationale for selecting fabrics. | Changes were made and information was added.  Since the manuscript was reviewed thoroughly and substantial changes were made, they are not copied here. |
| The discussion section is weak and does not consider findings from other studies but presents data from this study superficially (only one study cited in this section). | New information was added to the discussion section considering the study results and updated cited studies.  Since the manuscript was reviewed thoroughly and substantial changes were made, they are not copied here.  Importantly, this is an innovate idea and no previous studies were found on this specific subject. |
| **Reviewer2** |  |
| Regarding the Results section, why do you state that "Although the tests of the inner layer were focused on its thermal conductivity, all its tested thermal properties were included as additional information in this study. However, the discussion will focus on thermal conductivity only"? (line 200). Why focus on thermal conductivity only? | We agree with this comment. In fact, all the parameters were important.  What was written probably led to some misunderstanding.  Information was revised and changes were made to this topic. |
| I would suggest that a statement about the costs of the fabrics should be added to your discussion. During the discussion, I would suggest that you explicit the main limitations of your study and how this study can translate to clinical practice. | Information about the study limitations was added to the manuscript.  However, further studies will be conducted to address the other aspects (including the costs) of this innovative product. |
|  | We ask permission to change the title from  “Perioperative thermal insulation: testing comfort properties for different textile sets” to  “Development of a perioperative thermal insulation system: testing comfort properties for different textile sets”. |
|  | We aligned the correspondent author with the information provided in platform. |
| Please include your full ethics statement in the ‘Methods’ section of your manuscript file. In your statement, please include the full name of the IRB or ethics committee who approved or waived your study, as well as whether you obtained informed written or verbal consent. If consent was waived for your study, please include this information in your statement as well. | Ethical considerations was added into the methods.  This study has been approved by the Executive Board and by the Ethics Committee of the institution where the study was carried out (CEUOSSCMP/2014). |
| NOTE about data in Table 1 | In the previous manuscript, the data relating to the composition of the three fabrics were placed in 2 tables. After restructuring the data, information on fabric composition was placed in a single table (Table 1) |
| NOTE about data in Table 3 | In the previous manuscript, table 3 was composed of 8 tissue samples whose thermal properties had been tested. However, only 3 meshes were the focus of interest for testing, as they seem to have similar sensations of comfort to the touch.  Therefore, the other 5 samples were withdrawn from the study, to avoid information overload and bias. |
