# Supplementary material for: Development of a perioperative thermal insulation system: Testing comfort properties for different textile sets
Source: PLoS One. 2023 Sep 12;18(9):e0291424. doi: 10.1371/journal.pone.0291424 (PMC10497167; doi:10.1371/journal.pone.0291424)
Supplement: Attachment — Submitted filename: 18-01-2023Rebuttal_ letter.doc [file pone.0291424.s002.doc]

**Rebuttal letter**

Dear Professor Rajagopalan Parameshwaran,

Academic Editor of PLOS ONE

We are resubmitting the manuscript “Development of a perioperative thermal insulation system: testing comfort properties for different textile sets” for publication in PLOS ONE, according to instructions. We would like to thank you and the Reviewers for all the comments and suggestions on the manuscript and for the opportunity to review and resubmit this work. The comments and suggestions have enhanced the quality of this manuscript. This rebuttal letter shows the changes made and the explanation for each item. The changes and comments are highlighted in yellow.

| **Comments to the Author** |  |
| --- | --- |
| 5. Is the manuscript presented in an intelligible fashion and written in standard English?  PLOS ONE does not copyedit accepted manuscripts, so the language in submitted articles must be clear, correct, and unambiguous. Any typographical or grammatical errors should be corrected at revision, so please note any specific errors here. | The article was submitted for proofreading by a professional translator. |
| **Reviewer1** |  |
| P2L33: I would add blanket here so that it becomes clear what kind of textile you are talking about | “Blanket” added according to suggestion |
| P2L34: what is meant by "previous selection"? I would say that you just selected the mid and outer layers to investigate how the inner layer affects the thermal protection of the three-layered blanket? (goal of your study) | The expression “previous selection” was removed |
| P3L67: be more specific -> use blankets rather than system unless there are other structures to be considered. In this case, please mention all of them | “system” refers to the general classification when we want to define each type of protection (insulation system or warming system).  Examples of each type were added: “passive measures or thermal insulation systems, which create a barrier to prevent heat loss from the patient's body to the environment, such as cotton sheets or cotton blankets; and active measures or warming systems, which warm the patient's body through heat from external sources, such as water circulating mattresses and forced air warming blankets”. |
| P3L71ff: unclear sentence P4L74: one study demonstrated (not some authors demonstrated) | Sentence clarified according to suggestion, using “A study demonstrated…” |
| P4L77: be more precise with your terminology; what is meant by devices (active heating?); what is meant by systems (passive heating such as blankets?) | Text corrected: “warming systems” instead “warming devices”, because active warming are heating systems eg. circulating water mattresses and forced air warming blankets. |
| P4L79: what is the outcome of studies 15 and 16? | Information added:  In both studies, active measures (forced air warming systems and water circulating water mattresses) proved to be more effective in the controlling perioperative hypothermia than passive measures (cotton sheets). |
| P4L80: see comment P4L77 | Text corrected |
| P4L97ff: talk about layers; this section provides correct information about the 3-layered clothing system for outdoor activities. You do not need to provide that many details as this information is irrelevant to your study. | Details removed |
| P5L117: what is meant by "three fabrics (one layer each)" -> I think it is clear that a fabric is a single-layered material | “one layer each” has been removed from the manuscript |
| P5L119: selected -> please provide some information about why you previously decided on the mid and outer layer; what were the criteria for selection? -> this information might be provided in the methods section | The information about the criterion for selection of all layers has been added and replaced in the Method section “The fabrics for the mid and outer layers were chosen and tested based on maximum air permeability for the mid layer and impermeability for the outer layer.” |
| P6L126: this study investigates the effect of the inner layer of a thermal protective blanket on its thermal insulation performance... you do not need to elaborate on why it differs from sports clothing | Information about sports clothing has been removed. |
| P6L126f: why is the use and combination of existing fabrics distinguishing between sports clothing and your blanket? Sports clothing could also be made of existing fabrics… | The objective of the information was to reinforce that the study did not intend to develop new fabrics. Sentence changed “The blanket would be composed of existing fabrics, since developing new textile materials and fabrics is not an objective of this research” |
| P6L135f: unclear what is meant by "lower cost of the fabrics for testing." | The authors intended to spend minimum costs on the purchase of fabrics. But when revising the manuscript, this information was considered non-essential. Sentence has been removed. |
| P8L180: Please provide details for the Alambeta apparatus (producer, country) | Information has been provided.  “The tests were made using the Alambeta apparatus, Model SENSORA, developed by Liberec Register Company, from the Czech Republic” |
| P9L197: Please provide details for the thermal manikin (producer, country) | Details have been added.  “The thermal manikin used in this research, was manufactured by PT.Technic from Denmark, 2009” |
| P10L213: stability of manikin surface temperature? +/- 0.1°c? | Information has been added. 33⁰C (±0.1) |
| P10L213: range of temperature that the chamber can maintain (+/- 0.5°C?) | Information has been added. 22⁰C (±0.5) |
| P10L214: what do you mean by approximately? Give a range for the relative humidity during the experiment, e.g. 37-47% (as it was monitored continuously) | Information has been added. 42% (±2) |
| P10L224f: I would propose using the serial method only, as the manikin's body was not homogeneously covered with the blanket | Information related to Parallel method has been removed |
| P12L259ff: is this an aim of your study? | This is a variable of the method. |
| Table 3: Please provide SI-units for your parameters | SI-units have been added to Table 3 |
| Table 4: standard deviation for mean values? | Standard deviation for means of heat loss were added. |
| P16L335ff: omit this section; this is just a repetition of the paper by Kuklane et al. 2012 with no interpretation of your results | Section omitted |
| P17L358: however, the 3-layered blanket could further be optimized by considering adjustments in the middle and outer layer? | This topic was not considered since this is not an objective of the study. |
