# Supplementary material for: Development of a perioperative thermal insulation system: Testing comfort properties for different textile sets
Source: PLoS One. 2023 Sep 12;18(9):e0291424. doi: 10.1371/journal.pone.0291424 (PMC10497167; doi:10.1371/journal.pone.0291424)
Supplement: Attachment — Submitted filename: 19-08-2023Rebuttal_ letter.doc [file pone.0291424.s003.doc]

**Rebuttal letter**

Dear Professor Rajagopalan Parameshwaran,

Academic Editor of PLOS ONE

We are resubmitting the manuscript “Development of a perioperative thermal insulation system: testing comfort properties for different textile sets” for publication in PLOS ONE, according to the reviewers’ instructions. We would like to thank you and the Reviewers for all the comments and suggestions on the manuscript and for the opportunity to review and resubmit this work. The comments and suggestions have enhanced the quality of this manuscript. This rebuttal letter shows the changes made and the explanation for each item.

| **Comments to the Author** |  |
| --- | --- |
| **Reviewer1** |  |
| P2L35: as you are going to investigate the effect of the inner layer, there should be information provided in the abstract. Furthermore, it would be beneficial to know what kind of 2nd and 3rd layers you selected (e.g. if these are conventional fabrics used in perioperative thermal insulation systems) | The text was corrected, and some information is added to the abstract:  “Three fabric samples for the inner layer with same soft touch and different textile compositions were tested to find its effect on increasing the thermal insulation of the whole set, using a thermal manikin.”  “The best thermal insulation and thermal comfort performance was obtained by the set using an inner layer composed of polypropylene, polyamide, and elastane whose results were the highest thermal conductivity and thickness and the lowest maximum stationary heat flow density.”  “The results indicated that this fabric influenced positively the values of the whole set once increased its thermal protection.” |
| P5L102: the expression "three similar fabric samples" is not very scientific. Why are you going to investigate "similar" fabrics? You should be more interested in how differences between those fabrics affects the thermal insulation performance of the 3-layered blanket. What is the rationale you selected these three fabrics to be investigated? | The text was corrected:  “three fabric samples for the inner layer with same soft tactile sensation and different textile compositions were tested”  Information regarding the characteristics and criteria for selecting the inner layer fabrics has been provided within the “Material and Methods” section. |
| P5L106: unclear to which blanket concept you are referring to -> (passive) thermal insulation systems | Is clarified in the text that the blanket is a passive measure or a thermal insulation system. They are two different designations to the same concept.  “passive measures or thermal insulation systems, which create a barrier to prevent heat loss from the patient's body to the environment, such as cotton sheets or cotton blankets…”  “The blanket is a thermal insulation system because it creates a barrier to prevent heat loss from the patient's body to the environment.” |
| P5L112 do you refer to perspiration of the fabrics (e.g. substances released from the fabrics) or moisture management of sweat? | According to suggestion information is added, and it was clarified that the perspiration was to the patient´s sweat.  “although the fabrics used in extreme activities have technical properties such as breathability and perspiration, those characteristics would not be considered as important for this new solution since the humidity of the sweat produced by the person during surgery is incipient” |
| P5L118: it still does not become clear why the blanket concept should be based on "mountain clothing" -> there is no clear information provided in Figure 1 | To build a better passive thermal body protection system for patients in the perioperative context, the authors were inspired by the mountain clothing model. The figure 1 intended to show the scheme of the overlap of the fabrics. Some clarifications were added to the text before and after the Fig. 1 presentation, within the “Material and Methods” section. |
| P6L121: the inner layer is introduced in a general manner even though the study is about the investigation of the effect of the inner layer on total thermal protection. I would expect some more detailed information and explanations | Additional information and explanations regarding the inner layer have been included in the manuscript, within the “Material and Methods” section. |
| P10L203 I'm a bit astonished that you added exactly the temperature ranges I proposed for the manikin and the climatic chamber. I hope you did not just add them to the text, but they represent the accuracy of your devices. | Tests were conducted in an adiabatic chamber under controlled environmental conditions.  Variance: the range of values for the manikin´s temperature is 33ºC (±0.008), which was rounded to 0.01. Unfortunately, 0.1 was mistakenly used instead of 0.01.  The range of values for the relative humidity is 42% (±1.8) which was rounded to 2.  The range of room temperature values is correct: 22ºC (±0.5).  We would be pleased to provide the databases if You would like to consult them. |
|  | The figure has been changed. The reviewer is quite right to point out that an explanation of it would be necessary. However, we consider that the simpler figure is more adapted. |
